# Bimodal Genomic Approach Predicting Semaphorin 7A (SEMA7A) as Prognostic Biomarker in Adrenocortical Carcinoma

**DOI:** 10.3390/cancers17132078

**Published:** 2025-06-21

**Authors:** Anjali Dhall, Daiki Taniyama, Fathi Elloumi, Augustin Luna, Sudhir Varma, Suresh Kumar, Lauren Escobedo, Yilun Sun, Mirit I. Aladjem, Christophe E. Redon, Nitin Roper, William C. Reinhold, Jaydira Del Rivero, Yves Pommier

**Affiliations:** 1 Developmental Therapeutics Branch & Laboratory of Molecular Pharmacology, Center for Cancer Research, National Cancer Institute, National Institutes of Health, Bethesda, MD 20892, USA; anjali.dhall@nih.gov (A.D.); daiki.taniyama@nih.gov (D.T.); fathi.elloumi@nih.gov (F.E.); augustin.luna@nih.gov (A.L.); sudhir.varma@nih.gov (S.V.); suresh.kumar@nih.gov (S.K.); aladjemm@mail.nih.gov (M.I.A.); redonc@mail.nih.gov (C.E.R.); nitin.roper@nih.gov (N.R.); wcr@mail.nih.gov (W.C.R.); 2 Computational Biology Branch, National Library of Medicine, National Institutes of Health, Bethesda, MD 20892, USA; 3 HiThru Analytics, LLC, Princeton, NJ 08540, USA; 4 University of Maryland Marlene and Stewart Greenebaum Comprehensive Cancer Center, Baltimore, MD 21201, USA; lauren.escobedo@nih.gov (L.E.); yilun.sun@som.umaryland.edu (Y.S.)

**Keywords:** adrenocortical carcinoma (ACC), bimodal approach, gaussian mixture models, Semaphorin 7a (SEMA7A), immunohistochemistry (IHC)

## Abstract

Background: Adrenocortical carcinoma (ACC) is a rare and aggressive endocrine malignancy with a high mortality and poor prognosis. To elucidate the genetic underpinnings of ACCs, we have analyzed the transcriptome profiles of ACC tumor samples from patients enrolled in the TCGA and NCI cohorts. Methods: We developed a bimodal approach using Gaussian Mixture Models to identify genes with bimodal distribution in ACC samples. Among the 72 bimodally expressed genes that are used to stratify patients into prognostic groups, we focused on *SEMA7A*, as it encodes a glycosylphosphatidylinositol-anchored membrane glycoprotein (Semaphorin 7a) regulating integrin-mediated signaling, cell migration and immune responses. Results: Our findings reveal that high expression levels of *SEMA7A* gene are associated with poor prognosis (hazard ratio = 4.27; *p*-value < 0.001). In hormone-producing ACCs, *SEMA7A* expression is elevated and positively correlated with genes driving steroidogenesis, aldosterone and cortisol synthesis, including *CYP17A1*, *CYP11A1*, *INHA*, *DLK1*, *NR5A1* and *MC2R*. Correlation analyses show that *SEMA7A* is co-expressed with the *integrin-β1*, *FAK* (focal adhesion kinase) and *MAPK/ERK* (mitogen-activated protein kinase/extracellular signal regulated kinases) signaling pathways. Immunohistochemistry (IHC) staining demonstrates the feasibility of evaluating *SEMA7A* in ACC tissues and shows a significant correlation between gene expression (RNA-Seq) and protein expression (IHC). Conclusions: These findings suggest *SEMA7A* as a candidate for further research in ACC biology and a candidate for cancer therapy, as well as a potential prognosis biomarker for ACC patients.

## Simple Summary

Adrenocortical cancer (ACC) remains a challenging disease primarily due to the scarcity of reliable biomarkers for predicting patient outcomes and informing innovative therapeutic strategies, as well as its rarity, which restricts the scope of clinical trials. In our study, we developed a bimodal approach using RNA-seq data for stratifying cancer patients and predicting prognostic biomarkers. Our findings indicate that a substantial proportion of ACC tumors exhibit expression of SEMA7A, a glycoprotein involved in Semaphorin cell surface signaling. Notably, elevated levels of SEMA7A were identified as a poor prognostic biomarker and were associated with activation of the integrin–ERK-MAPK signaling pathways. These findings were further confirmed by an IHC analysis of ACC samples obtained from tissue microarray slides and National Cancer Institute (NCI) cancer patients. These results suggest that ACC tumors with high SEMA7A expression should be considered at elevated risk, and SEMA7A may serve as a potential target for immunotherapeutic strategies, including antibody–drug conjugates, T-cell engagers, and/or small-molecule inhibitors targeting the MAPK pathway.

## 1. Introduction

Adrenocortical cancer (ACC) is a rare and aggressive malignancy arising from the adrenal cortex with high morbidity and mortality rates. The estimated annual incidence of ACC is ~0.5 to 2 cases per million individuals worldwide. Notably, the incidence of ACC demonstrates a substantial age-related variation, with a peak occurring around the age of 50. In 40–60% of patients, ACCs typically exhibit an aggressive biological behavior with symptoms of hormone hyperproduction [1]. The prognosis for patients with ACC is generally poor, with a 5-year survival rate <15% among patients with distant metastases [2]. Prognosis is closely linked to the stage at which the disease is diagnosed, with earlier-stage presentations associated with more favorable outcomes [3,4]. The heterogeneous nature of ACC complicates its diagnosis and treatment, given the variability in clinical manifestations and prognostic outcomes [5]. The precise molecular mechanisms underpinning ACC are not fully elucidated despite significant advancements in genomic and transcriptomic profiling. Scientific breakthroughs are needed to identify diagnostic, prognostic, and therapeutic biomarkers, creating new opportunities for effective management strategies [6,7,8,9].

At present the clinical prognostic factors for ACC include tumor stage, cortisol secretion and patient age. Tumor staging serves as the principal determinant of prognosis, as well as the status of surgical resection margins. Histological features such as mitotic activity exceeding 20%, a Ki-67 index greater than 12% and a Weiss score greater than 6 have also been correlated with poor prognosis [10]. Given the disease’s heterogeneity and rarity, investigations have focused on assessing various immunohistochemistry markers to identify reliable prognostic factors. Efforts have also been made to develop genomic techniques for evaluating gene expression and alterations as potential molecular prognostic markers. Significant attention has been directed towards the Wnt/CTNNB1 and TP53 signaling pathways, which are frequently mutated in ACC, particularly with respect to CTNNB1 (beta-catenin) and P53 staining [11,12,13]. The presence of somatic CTNNB1 mutations has been associated with recurrence in ACC patients [14].

Systemic therapies are typically employed in the adjuvant setting or for patients exhibiting metastatic or unresectable disease. Mitotane, an adrenolytic agent, has remained the most utilized medication for over fifty years. It is administered as an adjuvant therapy following surgery or for inoperable or metastatic cases [15,16]. In cases of advanced disease that are not suitable for surgical intervention, the utilization of cytotoxic agents in conjunction with mitotane is indicated. Two commonly employed treatment regimens include the combination of etoposide, doxorubicin and cisplatin with mitotane (EDP-M), and the combination of streptozotocin with mitotane (S-M). These therapeutic approaches were evaluated in an international phase-III clinical trial. The findings indicated that the EDP-M regimen was associated with higher objective response rates (ORRs) and improved progression-free survival (PFS) when compared to the S-M regimen; however, no statistically significant difference in overall survival was observed [17]. Targeted therapies, including inhibitors of the insulin-like growth factor (IGF) pathway and immune checkpoint inhibitors, are currently under investigation in several clinical trials [18]. Preliminary findings indicate that these treatments may provide new options for patients with advanced ACC, though further research is necessary to fully establish their efficacy and safety profiles.

To address the current challenges, research endeavors are directed towards understanding the genetic underpinnings of ACC [19,20,21]. This step is crucial in the development of diagnostic and prognosis algorithms based on genomic data. Based on such approaches, several molecular characteristics have been identified, including prognostic markers such as (BUB1B, PINK1, MKI67) and molecular classification derived from the clustering of genomic profiles, which suggested the presence of two primary transcriptional clusters, designated as C1A/C1B, exhibiting a strong correlation with survival outcomes. However, the C1A/C1B classifier faces challenges in its implementation as a prognostic biomarker owing to its inherent complexity [5,21,22,23,24].

Alternatively, molecular profiling approaches based on bimodal gene expression have been used to identify biomarkers possessing distinct expression distributions, allowing for the classification of samples into two clearly defined expression states [25]. For instance, the estrogen receptor (*ESR1*) is a well-known bimodally expressed gene in breast cancer patients [26], and Schlafen 11 (*SLFN11*), a bimodal predictive genomic biomarker, predicts response to DNA-targeted chemotherapy [27]. In this study, we implemented a statistical approach to identify bimodally expressed genes using transcriptome profiles of 112 ACC tumor samples from both the TCGA database and from patients enrolled in NCI clinical trials. We report that a cell surface marker, Semaphorin 7a (*SEMA7A*), is significantly associated with poor prognosis, differentially up-regulated in ACC in comparison with normal adrenal tissues and reflects activation of the FAK and MAPK/ERK signaling pathways in a subset of ACC patients.

## 2. Material and Methods

### 2.1. Dataset Collection

In this study, we utilized transcriptomic and clinical data from the TCGA-ACC and NCI-ACC databases. For the NCI patient samples, a single institution study was conducted in accordance with recognized ethical guidelines as per The Belmont Reports and the Department of Health and Human Services Common Rule and was approved by the NIH Institutional Review Board. Written informed consent was obtained from all participants, and consent was approved by the NIH Institutional Review Board.

RNA-seq sequencing data processed using the NCI CCBR RNA-seq pipeline (https://github.com/skchronicles/RNA-seek.git (accessed on 6th April 2021)) and STAR (2.7.11b) to align reads to the hg38 reference genome. RSEM was used to normalize gene expression values expressed as log2(FPKM + 1) and the “RemoveBatchEffect” function from the “Limma” package was used to remove the impact of the library preparation protocols. In total, 112 ACC patients from TCGA-ACC (*n* = 79) and NCI-ACC (*n* = 33) were included in this study (see Table 1 and Table S1). Additionally, normalized RNA-seq expression data from multiple TCGA cancer types (including BLCA, BRCA, CESC, CHOL, ESCA, HNSC, KIRC, KIRP, LAML, LIHC, LUAD, LUSC, PAAD, PRAD, THCA, UCS and UVM) were obtained from cBioPortal and normal tissue expression datasets were obtained from the Genotype-Tissue Expression (GTEx) database.

### 2.2. Bimodal Approach

To identify RNA-seq expression bimodality we employed a combination of the Hartigan Dip test (using the R *Diptest* package, R version 4.2.3) and Normal/Gaussian Mixture Models (with the R *nor1mix* package using an Expectation–Maximization (EM) approach). For each individual gene, the expression distribution was sorted and tested for bimodality using the Dip test. If the distribution was found bimodal using Dip test with *p*-value < 0.05, we applied the *norMixEM* function from the R *nor1Mix* package to fit a mixture of two normal distributions. Once the fitting had converged, the parameters of this model, such as the means (*µ*_1_, *µ*_2_), weights (*w*_1_, *w*_2_ with *w*_1_ + *w*_2_ = 1), and standard deviation (*σ*_1_, *σ*_2_) for the two distributions, along with the log-likelihood, were computed per gene. We calculated an overall bimodal score, as a rough analog of a t-statistic, as the absolute difference in means divided by the larger of the two standard deviations (See Equation (1)). An additional criterion was that the difference between the weights for the two distributions was less than 0.25 to ensure the selection of genes with similar number of samples in the high and low expression distributions.

(1) $$Bimodal\;Score=\frac{\mathrm{abs}({µ}_{1}-{µ}_{2})}{\max(\sigma_{1},\sigma_{2})}$$

### 2.3. Statistical Analyses

Univariate and multivariate survival analyses were performed using the Cox proportional-hazards (Cox-PHs) regression model. Gene transcripts from ACC samples were stratified into two categories, high and low expression, based on the median expression cut-off. Significant differences in survival distributions between the high-risk (high expression) and low-risk (low expression) groups were assessed using the log-rank test, with the results presented in terms of Hazard Ratios (HRs), *p*-values, confidence interval and concordance index. An HR > 1 indicates a negative impact on patient survival, while HR < 1 improved survival. An HR of 1 indicates no effect on survival. The stratification of patients into high-risk and low-risk groups was visually represented using Kaplan–Meier (KM) survival curves, providing a distinct depiction of the survival probabilities over time for each group. We used the “survival”, “survminer” and “forestmodel” packages in R (version 4.2.3). Correlation analyses were performed using the Pearson correlation test. Mann–Whitney U test analyses were performed using GraphPad Prism 9.0 software (GraphPad Software Inc. (Boston, MA 02110, USA)).

### 2.4. TMA-Based SEMA7A IHC and Evaluation

Tissue microarray slide AG991 was used to stain ACC and normal adrenal gland tissue samples. Immunohistochemistry (IHC) staining was performed on LeicaBiosystems’ BondRX autostainer with the following conditions: Epitope Retrieval 2 (EDTA) 20′, SEMA7A (Santa Cruz #sc-374432, 1:100 incubated 30′), and the Bond Polymer Refine Detection Kit (LeicaBiosystems #DS9800 (Buffalo Grove, IL, USA)). Isotype control reagent (mouse IgG2a, BD Biosciences #553454) was used in place of primary antibody for the negative control. Slides were removed from the Bond autostainer, dehydrated through ethanol, cleared with xylene and coverslipped. Slides were scanned using a Leica Aperio AT2 scanner (Leica Biosystems, Buffalo Grove, IL, USA) at 20× magnification. The stained IHC slides were quantified using the HALO image analysis software 3.6 (Indica Labs, Albuquerque, NM, USA). Automated quantification of percent positive cells for each core was performed using cytonuclear algorithm version 2.0.5.

### 2.5. NCI-ACC Patient Specimens

For the NCI-ACC patient specimens, formalin-fixed paraffin-embedded tissue sections were used for IHC. SEMA7A IHC was performed using a previously validated method [28]. Optimized staining protocol included antigen retrieval performed by microwave heating in Antigen Retrieval Buffer (pH 6.0) (#ab93678, Abcam (Abcam Inc. 152 Grove Street, Waltham, MA, USA)) for 20 min. The sections were incubated with mouse monoclonal anti-SEMA7A antibody (Santa Cruz #sc-374432, 1:100 dilution in TBST (1 × TBS + 0.5% Tween 20)) overnight at 4 °C. The sections were then incubated for 30 min in N-histofine Simple Stain MAX PO(MULTI) (Nichirei Biosciences Inc., # NIC-414151F (Nichirei Higashi-Ginza Building, 6-19-20, Tsukiji, Chuo-ku, Tokyo 104-8402, Japan)) followed by 5 min incubation with chromogen DAB-3S (original dilution by manufacturer, Nichirei Biosciences Inc.; #415192F (Nichirei Higashi-Ginza Building, 6-19-20, Tsukiji, Chuo-ku, Tokyo 104-8402, Japan)) at room temperature.

## 3. Results

### 3.1. Overall Workflow and Implementation of the Bimodal Approach

**Figure 1:**
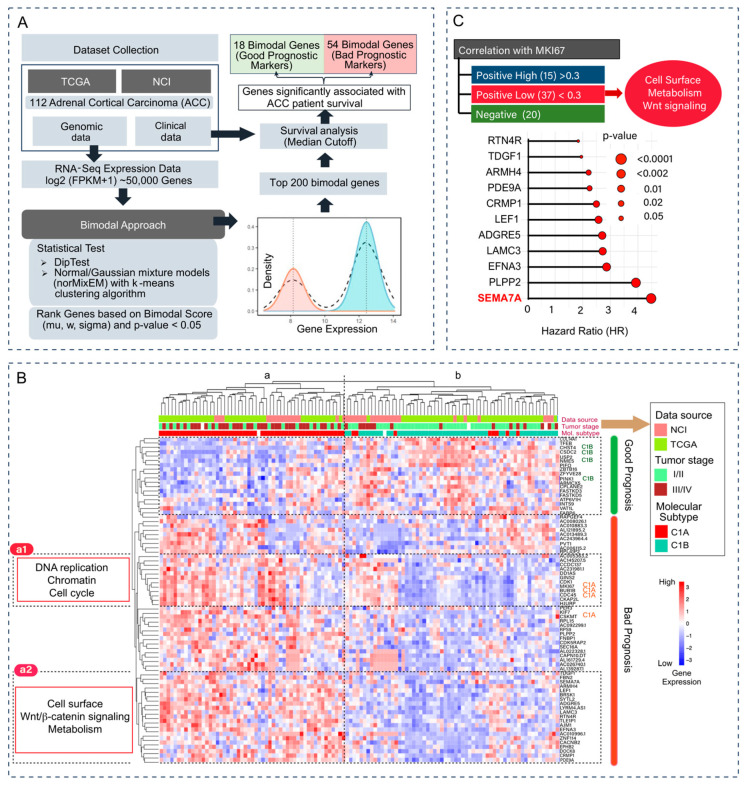
(**A**) Study architecture and bimodal gene selection. (**B**) Unsupervised hierarchical clustering heatmap displaying 72 bimodal genes associated with ACC patient survival, tumor stage, molecular subtype (C1A/C1B), and *PINK1*, *BUB1B*, *MKI67* signature. (**C**) Classification of the 72 bimodal genes into 3 groups based on correlation analysis with *MKI67*. Subgroup **a1** is enriched for DNA replication markers and subgroup **a2** for cell surface markers including *SEMA7A*.

The overall workflow for the selection of bimodal genes is provided in Figure 1A. At first, we applied the bimodal algorithm to the gene expression data collected from both TCGA and NCI. Ranking the genes based on their bimodal scores led to the selection of 200 genes with the highest bimodal distribution. Univariate survival analysis on those top 200 genes retrieved 72 genes significantly associated with the survival of ACC patients. Among them 54 are significantly associated with poor prognosis, and 18 with better overall survival (Figure 1A). The list of the 72 bimodal-survival genes is provided in (Table S2). To evaluate the robustness of these associations, we performed multivariate survival analysis while adjusting for potential confounding clinical variables, including age, tumor stage, and gender. This analysis confirmed that 56 out of the 72 bimodal genes retained a statistically significant association with overall survival in ACC patients (Table S3 and Figure S2). We also assigned the NCI-ACC patients to C1A/C1B molecular subtypes using the ConsensusClusterPlus approach previously used for the TCGA-ACC samples [19,29] (Figure 1B, upper section labeled as molecular subtypes in red for C1A and blue for C1B).

Next, we computed a double clustering heatmap displaying both patient samples (columns in Figure 1B) and expression of the 72 bimodal genes (rows). Patient distribution formed two main clusters (labeled “a” and “b” in Figure 1B). Cluster “**a**” is enriched for patient with advanced tumors (stage III and IV) and C1A subtype while it is the reverse for patients in cluster “**b**” (high fraction of localized tumors with C1B staging). Analysis of the gene distribution (rows in Figure 1B) shows an upper cluster encompassing genes that tend to be highly expressed in the cluster “**b**” patients and can be considered good prognosis genes. Consistently, among them, four are known C1B genes: *CHST4* (encoding CarboHydrate sulfotransferase 4), *CSDC2* (encoding Cold Shock Domain Containing C2), *NME5* (encoding Non-Metastasis cell 5 protein) and *PINK1* (encoding PTEN-Induced Kinase 1, whose high expression are well-established good prognosis biomarkers in ACC [21,29]. The bimodal survival genes associated with poor prognosis (cluster “a” in Figure 1B), are distributed in four clusters. Two of them are annotated as “a1” and “a2” in Figure 1B. Cluster “**a1**” include genes associated with DNA replication, chromatin and the cell cycle: *HJURP*, *CDK1*, *CDCA5*, *GINS2*, *DDIAS*. Cluster “**a2**” includes cell surface markers, metabolism and Wnt-signaling-related genes including *PDE9A*, *BRSK1*, *SYTL2*, *RTN4R*, *EFNA3*, *PLPP2*, *CRMP1*, *LAMC3*, *LEF1* and *SEMA7A*.

Further analyses were performed to divide the bimodal genes based on their relationship with *MKI67* and identify novel predictive biomarkers that would complement *MKI67*. Thirty-four genes showed a low correlation with *MKI67* (Figure 1C). Among them *SEMA7A*, which encodes the cell surface marker Semaphorin 7A showed the highest Hazard Ratio (HR = 4.27 and significant *p*-value < 0.001) (Figure 1C). These results reveal SEMA7A expression as a novel prognosis biomarker with a broad bimodal expression in ACC.

### 3.2. High Expression of SEMA7A in ACC

**Figure 2:**
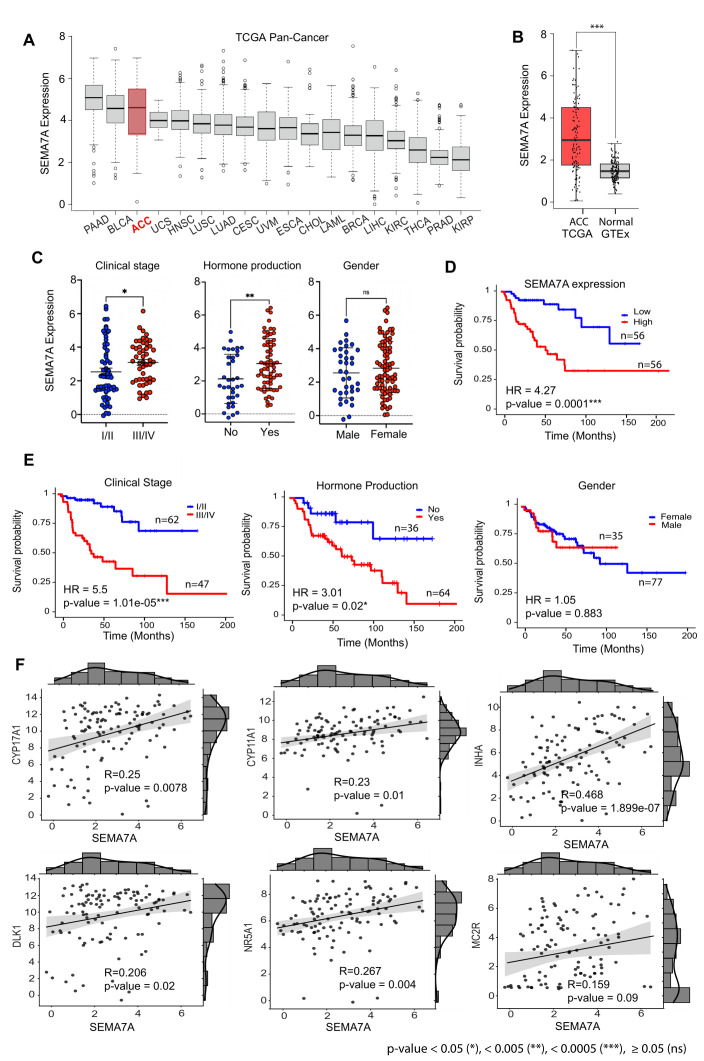
Expression of *SEMA7A* in ACC and SEMA7A expression as prognosis biomarker in ACC. (**A**) *SEMA7A* expression in various TCGA cancer types. (**B**) Differential expression and bimodal distribution of *SEMA7A* in normal adrenal gland (GTEx) and ACC. (**C**) Expression of *SEMA7A* as a function of tumor stage (III/IV vs. stage I/II), hormone production and gender of ACC patients. (**D**,**E**) Kaplan–Meier survival plots demonstrating the significant association of *SEMA7A* expression with clinical stage and hormone production in ACC patients. (**F**) Scatter plot shows correlation between SEMA7A expression (x-axis) with steroidogenesis-related genes (y-axis) for individual ACC sample.

In the TCGA-pan cancer datasets, ACC is with pancreatic adenocarcinoma (PAAD) and bladder cancer (BLCA) among the cancers expressing the highest levels of *SEMA7A* (Figure 2A). This high expression is specific to ACC patients as the expression of *SEMA7A* is low in normal adrenal gland (Figure 2B and Figure S1). The normal tissues expressing the highest levels of *SEMA7A* are lymphoid, nervous and germinal (Figure S1). We next examined whether *SEMA7A* expression is associated with clinical stage, hormone production and gender. High expression of SEMA7A was observed in high-grade (III/IV) and hormone-producing ACC (Figure 2C). Female patients tend to have higher SEMA7A expression than males, but the difference is not significant (Figure 2C). The prognosis value of SEMA7A is shown in Figure 2D with significantly reduced survival of patients with high *SEMA7A* expression (Hazard Ratio (HR) = 4.27 and *p*-value < 0.001). As expected, stages III/IV and hormone-producing tumors are also at significantly higher risk (Figure 2E). No significant difference was observed between males and female patients (Figure 2E). Correlation analyses showed a significant association of *SEMA7A* expression with the expression of adrenocorticotropic hormone receptor, steroidogenic enzymes, cholesterol transporters and their transcriptional regulator genes (*CYP11A1*, *CYP17A1*, *MC2R*, *NR5A1/SF1*, *DLK1* and *INHA*) [30,31,32,33,34,35] (Figure 2F).

### 3.3. Activation of the SEMA7A-Integrin-β1 Downstream Signaling Pathways in ACC

**Figure 3:**
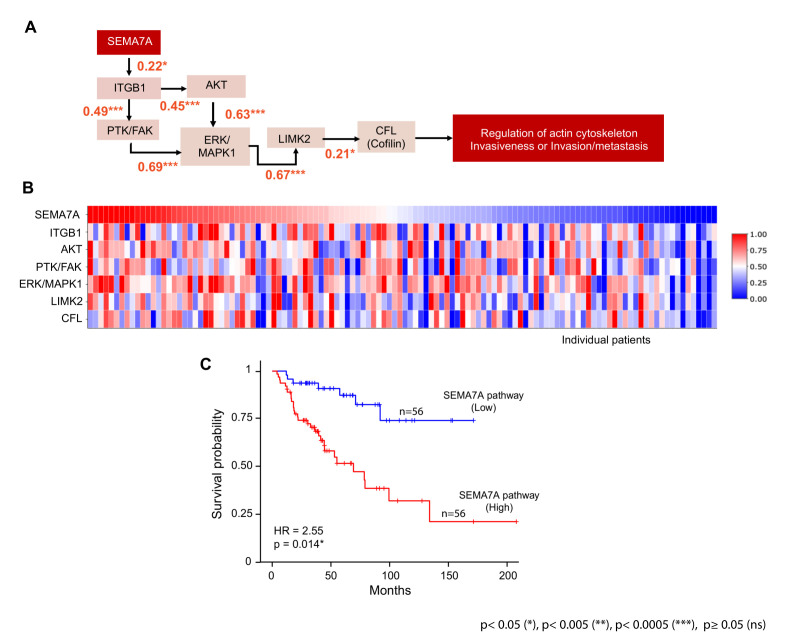
(**A**) MAPK/ERK signaling pathway is co-regulated via SEMA7A. (**B**) Heatmap shows the expression of neighboring genes of MAPK/ERK pathway in ACC patients. (**C**) Kaplan–Meier survival plot, based on average expression of SEMA7A pathway genes, divided into two groups based on median expression cut-off.

SEMA7A is known to belong to a SEMA7A-integrin-β1 axis that activates Mitogen-Activated Protein Kinase (MAPK) cascades, especially the ERK/MAPK signaling pathway, which plays crucial role in regulating oncogenic functions, including metastasis and tumor progression [36,37,38,39]. As shown in Figure 3A, expression of the downstream pathway genes *ITGB1*, *AKT*, *PTK/FAK*, *ERK/MAPK1*, *LIMK1*, *CFL1* shows significant positive correlation with *SEMA7A* expression and with each other. These observations are consistent with the functional activation of the downstream pathways of SEMA7A in the ACC tumors expressing high *SEMA7A* transcripts.

An expression heatmap of SEMA7A-integrin-β1 downstream pathway genes stratifies the ACC patient samples into two subgroups, one having high expression and the other having low expression of those genes (Figure 3B). To explore the association between the downstream genes of the SEMA7A-integrin-β1 pathway and overall survival in ACC patients, we calculated the average expression of genes involved in the SEMA7A pathway and stratified the patients into two groups based on the median expression value (cut-off). The survival analysis shows the poor prognosis of patients having high average expression of SEMA7A pathway genes (Figure 3C).

Together these results demonstrate the functionality of the SEMA7A pathway in a significant fraction of ACC tumors and the significantly poor prognosis of patients with high *SEMA7A* expression and SEMA7A pathway activation.

### 3.4. Exploration of SEMA7A Protein Expression in ACC

**Figure 4:**
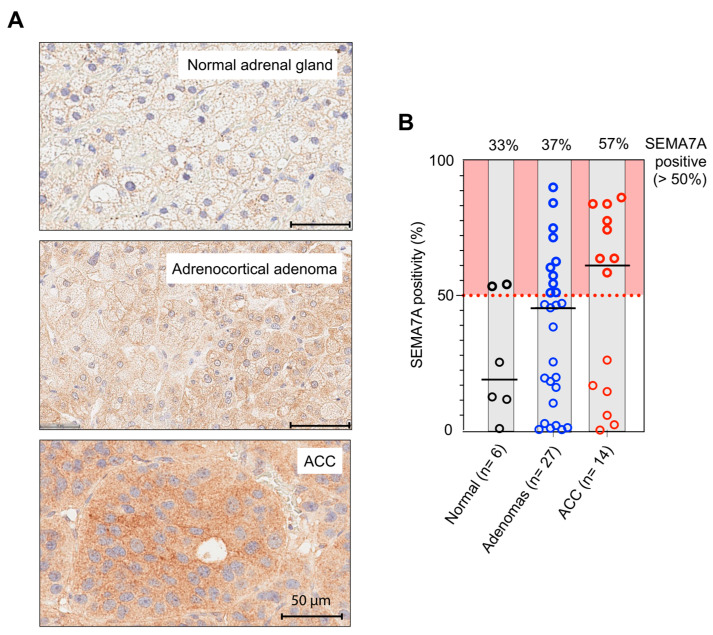
(**A**) Immunohistochemistry (IHC) examination of SEMA7A protein expression in normal adrenal gland tissues, adrenocortical adenomas (benign) and adrenocortical carcinomas (ACC). (**B**) Percentage of SEMA7A-positive cases in normal, benign and malignant tissue samples. Note the bimodal distribution of SEMA7A in ACC.

To compare SEMA7A transcripts and protein expression, we first used tissue microarray slides to determine whether SEMA7A protein expression could be measured in normal adrenal gland (*n* = 6), adrenocortical adenoma (*n* = 27) and ACC (*n* = 14) tumor tissues. Figure 4A shows representative immunohistochemistry (IHC) staining images demonstrating the fraction of cells expressing SEMA7A in ACC tissues compared to normal adrenal gland tissue and adrenocortical adenoma (benign tumor). Quantification (Figure 4B) is consistent with the bimodal distribution of SEMA7A expression observed at the transcript level. Indeed, some samples show a very high percentage of positive cells expressing SEMA7A while other samples display low fractions. This suggests that SEMA7A protein levels determined by IHC can potentially be used as a biomarker to identify specific subgroups of ACC patients and score tumor aggressiveness instead of or in conjunction with RNA-Seq analyses.

**Figure 5:**
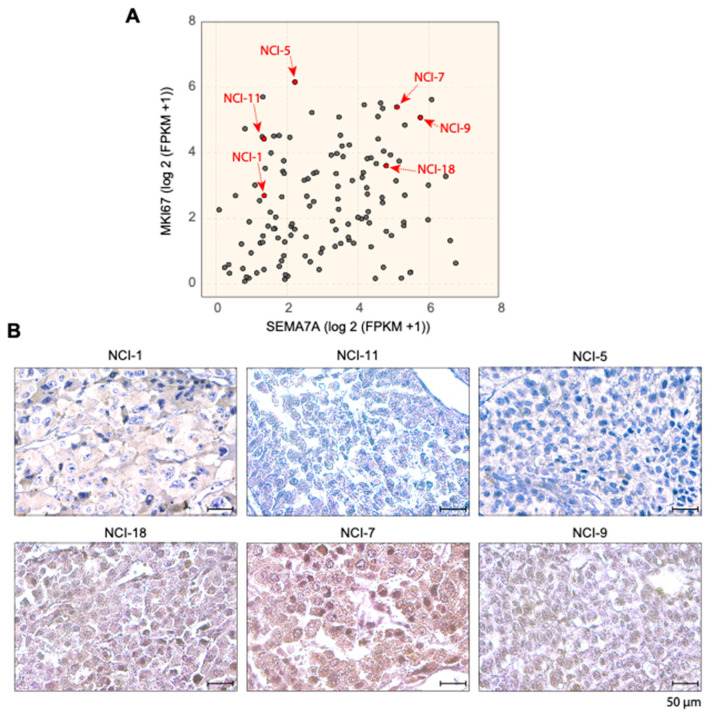
(**A**) Wide distribution of SEMA7A expression measured by RNA-Seq in 112 ACC tumor samples. (**B**) Representative immunohistochemistry (IHC) staining of SEMA7A in three samples with high vs. low expression of SEMA7A in NCI-ACC patients.

To validate the IHC approach in ACC tumor samples, we compared the protein and RNA expression levels of SEMA7A in the NCI-ACC samples. We selected three samples with low and three with high SEMA7A RNA-Seq expression levels. As shown in Figure 5, the samples with low RNA-Seq levels do not show high expression at the protein. By contrast, patients with high RNA-seq expression of SEMA7A (log2(FPKM + 1) (≥5) show protein expression. These results indicate a positive correlation between RNA-Seq expression and protein levels in the selected ACC samples and suggest the value of measuring SEMA7A using both RNA-Seq and IHC to classify ACC patients.

## 4. Discussion

In this study, we have implemented a combination of the **Hartigan Dip test** and **Gaussian Mixture Modeling (GMM)** using the *nor1mix* package, with the EM algorithm. This hybrid bimodal approach utilizes non-parametric testing for the screening of unimodality, and parametric testing to quantify RNA-seq expression bimodality. In the literature several other methods have been proposed to detect the bimodal gene expression [26,40,41], for instance, the Bimodal Index (BI) approach for selecting and ranking bimodal genes; based on a two-component gaussian mixture model with equal variances, BI captures both the standardized distance between the means of the two distributions and the proportion of samples in each cluster [42]. Tibshirani et al. proposed an **outlier-oriented method** named Cancer Outlier Profile Analysis (COPA) which detects outlier-sum statistic flag genes in which a subset of samples show unusually high expression, capturing both isolated and recurrent outliers [43]. In addition, clustering based approaches use unsupervised learning to divide data into two or more groups based on k-means, hierarchical clustering (non-parametric methods) making no assumptions about the data, whereas model-based clustering (parametric method) assumes the data comprise a distribution that is a mixture of components. The number of components can be explained by any distribution via density function. Further, the one-component-based model is compared with a mixture of two-component models to obtain the bimodal gene expression in microarray or next-generation sequencing data. Justino et al. introduced an integrated method to identify bimodal genes across multiple TCGA datasets [44]. However, their analysis did not include ACC samples [26,44,45]. Our aim was to explore a novel approach to classify ACC patients and capture heterogeneity across ACC tumors.

ACC presents considerable challenges due to its rarity, the complexity of its biology and its poor prognosis. Furthermore, due to the restricted number of cell lines and preclinical models, ACC patients have limited treatment options. Surgical resection continues to be the foremost approach for the treatment of localized cases, while a metastasectomy has been proposed for the management of recurrent or metastatic ACC. This strategy carries significant therapeutic and palliative implications and is only associated with prolonged survival in carefully selected patients. Due to the prevalence of surgical interventions, high quality samples for biological and genomic analyses are routinely available at the Center for Cancer Research (CCR) of the National Cancer Institute at the National Institutes of Health (see Figure 1 and Figure 5).

To identify novel biomarkers for ACC, we have developed a method to identify bimodally expressed genes in ACC patient tumors to stratify patients based on their overall survival and discover predictive biomarkers. At first, we used RNA-seq expression profiles of ACC patient tumors samples and computed the bimodal score for each gene. We found 72 genes that had a significant association with overall survival. Among them, SEMA7A came up as the gene with the significant association with prognosis independently of *MKI67* (see Figure 5A). In our analysis, we have observed that *SEMA7A* is significantly up-regulated in ACC as compared to normal adrenal gland tissues (see Figure 2). *SEMA7A* belongs to the Semaphorin gene family. It encodes SEMA7A (CDw108), a signaling glycoprotein (as its name indicates) anchored to the cell surface via glycosylphosphatidylinositol (GPI) linkage [46]. Upon cleavage of the GPI membrane anchor, SEMA7A exists as both shed and membrane bound. SEMA7A influences cellular dynamics in both cell-autonomous and non-autonomous manners. It enhances the mobility of melanocytes, neuronal cells and immune cells through the activation of β-1 integrin signaling pathways [37,38,47]. In its normal physiological role, SEMA7A binds β1-integrin to activate downstream signaling cascades, including the pro-invasive MAPK/ERK and pro-survival PI3K/AKT pathways [48].

Recent findings have identified SEMA7A as a tumor promoter, accelerating tumor growth, increasing invasiveness, and promoting adhesion to tumor-associated extracellular matrices. SEMA7A induces epithelial to mesenchymal transition, and promoting metastasis in melanoma, glioblastoma, oral, kidney and breast cancers [36,49,50,51,52,53]. Moreover, high expression of SEMA7A has been associated with significantly decreased patient survival in ER + breast cancer patients [54]. Prior to this work, no associations between SEMA7A and ACC have been observed. Relevant to our study, in neuronal cells of the hypothalamus, SEMA7A expression is regulated by steroid hormones [55], an association we found also in ACC (see Figure 2). SEMA7A is also upregulated in high-grade (Stage III/IV) and hormone-producing ACC patients (see Figure 2). Our correlation analyses show that the SEMA7A-integrin-β1 axis activates the ERK/MAPK signaling pathway in a subset of ACC patients with poor prognosis (see Figure 3).

Immunohistochemistry (IHC) staining of ACC tissues shows a bimodal distribution of SEMA7A in ACC patients with lower levels in normal adrenal tissues and benign adrenal tumors (see Figure 4 and Figure 5) and high protein levels in ACC. The positive correlation between RNA-seq and immunohistochemistry (IHC) in NCI-ACC patient samples suggests that SEMA7A could be a promising prognostic biomarker for clinical applications.

## 5. Conclusions

Our study reports a bimodal gene detection approach to identify clinically relevant biomarkers in adrenocortical carcinoma patients. Univariate and multivariate analyses show that the bimodally expressed gene SEMA7A acts as a poor prognostic biomarker in ACC. Correlation analyses also reveal that SEMA7A-integrin-β1 activates the MAPK signaling pathway in ACC patients. Over-expression of SEMA7A at both the transcriptomic and protein levels highlight it as a potential biomarker for further investigations and for application in clinical diagnostics, particularly for forecasting outcomes in ACC patients. Finally, SEMA7A emerges as a potential therapeutic target for the development of immunotherapies, including antibody drug conjugates (ADCs) and T-cell engagers, for the treatment of ACC.

## Figures and Tables

**Table 1:** Clinical and demographic characteristics of ACC patients.

Variable	Number of Patients (Total = 112)
**Age**	
Median age <49 years	56
Median age ≥49 years	56
**Gender**	
Female	77
Male	35
**Tumor Stage**	
Stage I	12
Stage II	50
Stage III	18
Stage IV	29
Not Available	3
**Vital Status**	
Alive	75
Dead	37
**Median overall Survival (OS)**	
<43 months	58
≥43 months	54
**Hormone Producing**	
Yes	64
No	35
Not Available	13

## Data Availability

The clinical information for NCI and TCGA ACC patients is provided in Supplementary Table S1 and list of 72 genes is provided in Supplementary Table S2. Univariate and multivariate survival analysis results are included in Supplementary Tables S2 and S3. The analysis code and normalized log2(FPKM + 1) expression data matrix is provided in Zenodo repository: https://doi.org/10.5281/zenodo.15253151.

## Supplementary Materials

The following are available online at https://www.mdpi.com/article/10.3390/cancers17132078/s1, Figure S1: Distribution of SEMA7A expression across a wide range of normal human tissues obtained from GTEx database, Figure S2: Forest plot of multivariate survival analysis of bimodal genes, adjusted for clinical covariates (Age, tumor stage and gender), Table S1: Clinical and demographic features of patients with adrenocortical carcinoma (ACC), Table S2: Univariate survival analysis of 72 bimodal prognostic markers, corresponding gene annotations, Hazard ratio and p-value, Table S3: Multivariate survival analysis of 72 bimodal genes. The analysis is adjusted for known clinical confounders incliuding (Age, Gender and Tumor Stage).

## References

[1] Else T., Kim A.C., Sabolch A., Raymond V.M., Kandathil A., Caoili E.M., Jolly S., Miller B.S., Giordano T.J., Hammer G.D. (2014). Adrenocortical carcinoma. Endocr. Rev..

[2] Libe R., Huillard O. (2023). Adrenocortical carcinoma: Diagnosis, prognostic classification and treatment of localized and advanced disease. Cancer Treat. Res. Commun..

[3] Sidhu S., Sywak M., Robinson B., Delbridge L. (2004). Adrenocortical cancer: Recent clinical and molecular advances. Curr. Opin. Oncol..

[4] Fassnacht M., Allolio B. (2009). Clinical management of adrenocortical carcinoma. Best. Pract. Res. Clin. Endocrinol. Metab..

[5] Lam A.K. (2021). Adrenocortical Carcinoma: Updates of Clinical and Pathological Features after Renewed World Health Organisation Classification and Pathology Staging. Biomedicines.

[6] Cheng Y., Kou W., Zhu D., Yu X., Zhu Y. (2021). Future Directions in Diagnosis, Prognosis and Disease Monitoring of Adrenocortical Carcinoma: Novel Non-Invasive Biomarkers. Front. Endocrinol..

[7] Mizdrak M., Ticinovic Kurir T., Bozic J. (2021). The Role of Biomarkers in Adrenocortical Carcinoma: A Review of Current Evidence and Future Perspectives. Biomedicines.

[8] Marquardt A., Landwehr L.S., Ronchi C.L., di Dalmazi G., Riester A., Kollmannsberger P., Altieri B., Fassnacht M., Sbiera S. (2021). Identifying New Potential Biomarkers in Adrenocortical Tumors Based on mRNA Expression Data Using Machine Learning. Cancers.

[9] Mohan D.R., Lerario A.M., Else T., Mukherjee B., Almeida M.Q., Vinco M., Rege J., Mariani B.M.P., Zerbini M.C.N., Mendonca B.B. (2019). Targeted Assessment of G0S2 Methylation Identifies a Rapidly Recurrent, Routinely Fatal Molecular Subtype of Adrenocortical Carcinoma. Clin. Cancer Res..

[10] Dinnes J., Bancos I., Ferrante di Ruffano L., Chortis V., Davenport C., Bayliss S., Sahdev A., Guest P., Fassnacht M., Deeks J.J. (2016). MANAGEMENT OF ENDOCRINE DISEASE: Imaging for the diagnosis of malignancy in incidentally discovered adrenal masses: A systematic review and meta-analysis. Eur. J. Endocrinol..

[11] Tsai W.H., Dai S.H., Lee C.C., Chien M.N., Zeng Y.H. (2023). A Clinicopathological Analysis of Asian Patients with Adrenocortical Carcinoma: A Single-Center Experience. Curr. Oncol..

[12] Ragazzon B., Libe R., Gaujoux S., Assie G., Fratticci A., Launay P., Clauser E., Bertagna X., Tissier F., de Reynies A. (2010). Transcriptome analysis reveals that p53 and beta-catenin alterations occur in a group of aggressive adrenocortical cancers. Cancer Res..

[13] Waldmann J., Patsalis N., Fendrich V., Langer P., Saeger W., Chaloupka B., Ramaswamy A., Fassnacht M., Bartsch D.K., Slater E.P. (2012). Clinical impact of TP53 alterations in adrenocortical carcinomas. Langenbecks Arch. Surg..

[14] Gaujoux S., Grabar S., Fassnacht M., Ragazzon B., Launay P., Libe R., Chokri I., Audebourg A., Royer B., Sbiera S. (2011). beta-catenin activation is associated with specific clinical and pathologic characteristics and a poor outcome in adrenocortical carcinoma. Clin. Cancer Res..

[15] Terzolo M., Angeli A., Fassnacht M., Daffara F., Tauchmanova L., Conton P.A., Rossetto R., Buci L., Sperone P., Grossrubatscher E. (2007). Adjuvant mitotane treatment for adrenocortical carcinoma. N. Engl. J. Med..

[16] Terzolo M., Fassnacht M., Perotti P., Libe R., Kastelan D., Lacroix A., Arlt W., Haak H.R., Loli P., Decoudier B. (2023). Adjuvant mitotane versus surveillance in low-grade, localised adrenocortical carcinoma (ADIUVO): An international, multicentre, open-label, randomised, phase 3 trial and observational study. Lancet Diabetes Endocrinol..

[17] Fassnacht M., Terzolo M., Allolio B., Baudin E., Haak H., Berruti A., Welin S., Schade-Brittinger C., Lacroix A., Jarzab B. (2012). Combination chemotherapy in advanced adrenocortical carcinoma. N. Engl. J. Med..

[18] Pegna G.J., Roper N., Kaplan R.N., Bergsland E., Kiseljak-Vassiliades K., Habra M.A., Pommier Y., Del Rivero J. (2021). The Immunotherapy Landscape in Adrenocortical Cancer. Cancers.

[19] Zheng S., Cherniack A.D., Dewal N., Moffitt R.A., Danilova L., Murray B.A., Lerario A.M., Else T., Knijnenburg T.A., Ciriello G. (2016). Comprehensive Pan-Genomic Characterization of Adrenocortical Carcinoma. Cancer Cell.

[20] Mohan D.R., Borges K.S., Finco I., LaPensee C.R., Rege J., Solon A.L., Little D.W., Else T., Almeida M.Q., Dang D. (2023). beta-Catenin-Driven Differentiation Is a Tissue-Specific Epigenetic Vulnerability in Adrenal Cancer. Cancer Res..

[21] Assie G., Jouinot A., Fassnacht M., Libe R., Garinet S., Jacob L., Hamzaoui N., Neou M., Sakat J., de La Villeon B. (2019). Value of Molecular Classification for Prognostic Assessment of Adrenocortical Carcinoma. JAMA Oncol..

[22] Morimoto R., Satoh F., Murakami O., Suzuki T., Abe T., Tanemoto M., Abe M., Uruno A., Ishidoya S., Arai Y. (2008). Immunohistochemistry of a proliferation marker Ki67/MIB1 in adrenocortical carcinomas: Ki67/MIB1 labeling index is a predictor for recurrence of adrenocortical carcinomas. Endocr. J..

[23] Almeida M.Q., Bezerra-Neto J.E., Mendonca B.B., Latronico A.C., Fragoso M. (2018). Primary malignant tumors of the adrenal glands. Clinics.

[24] Zhang F., Zhang F., Liu Z., Wu K., Zhu Y., Lu Y. (2019). Prognostic Role of Ki-67 in Adrenocortical Carcinoma After Primary Resection: A Retrospective Mono-Institutional Study. Adv. Ther..

[25] Ertel A. (2010). Bimodal gene expression and biomarker discovery. Cancer Inform..

[26] Moody L., Mantha S., Chen H., Pan Y.X. (2019). Computational methods to identify bimodal gene expression and facilitate personalized treatment in cancer patients. J. Biomed. Inform..

[27] Murai J., Thomas A., Miettinen M., Pommier Y. (2019). Schlafen 11 (SLFN11), a restriction factor for replicative stress induced by DNA-targeting anti-cancer therapies. Pharmacol. Ther..

[28] Takashima T., Sakamoto N., Murai J., Taniyama D., Honma R., Ukai S., Maruyama R., Kuraoka K., Rajapakse V.N., Pommier Y. (2021). Immunohistochemical analysis of SLFN11 expression uncovers potential non-responders to DNA-damaging agents overlooked by tissue RNA-seq. Virchows Arch..

[29] de Reynies A., Assie G., Rickman D.S., Tissier F., Groussin L., Rene-Corail F., Dousset B., Bertagna X., Clauser E., Bertherat J. (2009). Gene expression profiling reveals a new classification of adrenocortical tumors and identifies molecular predictors of malignancy and survival. J. Clin. Oncol..

[30] Chida D., Nakagawa S., Nagai S., Sagara H., Katsumata H., Imaki T., Suzuki H., Mitani F., Ogishima T., Shimizu C. (2007). Melanocortin 2 receptor is required for adrenal gland development, steroidogenesis, and neonatal gluconeogenesis. Proc. Natl. Acad. Sci. USA.

[31] Ferraz-de-Souza B., Lin L., Achermann J.C. (2011). Steroidogenic factor-1 (SF-1, NR5A1) and human disease. Mol. Cell Endocrinol..

[32] Rubtsov P., Karmanov M., Sverdlova P., Spirin P., Tiulpakov A. (2009). A novel homozygous mutation in CYP11A1 gene is associated with late-onset adrenal insufficiency and hypospadias in a 46,XY patient. J. Clin. Endocrinol. Metab..

[33] Sigala S., Bothou C., Penton D., Abate A., Peitzsch M., Cosentini D., Tiberio G.A.M., Bornstein S.R., Berruti A., Hantel C. (2022). A Comprehensive Investigation of Steroidogenic Signaling in Classical and New Experimental Cell Models of Adrenocortical Carcinoma. Cells.

[34] Ansell P.J., Zhou Y., Schjeide B.M., Kerner A., Zhao J., Zhang X., Klibanski A. (2007). Regulation of growth hormone expression by Delta-like protein 1 (Dlk1). Mol. Cell Endocrinol..

[35] Sun N.Y., Kumar S., Kim Y.S., Varghese D., Mendoza A., Nguyen R., Roper N. (2024). Identification of DLK1, a Notch ligand, as an immunotherapeutic target and regulator of tumor cell plasticity and chemoresistance in adrenocortical carcinoma. bioRxiv.

[36] Song Y., Wang L., Zhang L., Huang D. (2021). The involvement of semaphorin 7A in tumorigenic and immunoinflammatory regulation. J. Cell Physiol..

[37] Pasterkamp R.J., Peschon J.J., Spriggs M.K., Kolodkin A.L. (2003). Semaphorin 7A promotes axon outgrowth through integrins and MAPKs. Nature.

[38] Suzuki K., Okuno T., Yamamoto M., Pasterkamp R.J., Takegahara N., Takamatsu H., Kitao T., Takagi J., Rennert P.D., Kolodkin A.L. (2007). Semaphorin 7A initiates T-cell-mediated inflammatory responses through alpha1beta1 integrin. Nature.

[39] Garcia-Areas R., Libreros S., Amat S., Keating P., Carrio R., Robinson P., Blieden C., Iragavarapu-Charyulu V. (2014). Semaphorin7A promotes tumor growth and exerts a pro-angiogenic effect in macrophages of mammary tumor-bearing mice. Front. Physiol..

[40] Tong P., Chen Y., Su X., Coombes K.R. (2013). SIBER: Systematic identification of bimodally expressed genes using RNAseq data. Bioinformatics.

[41] Bessarabova M., Kirillov E., Shi W., Bugrim A., Nikolsky Y., Nikolskaya T. (2010). Bimodal gene expression patterns in breast cancer. BMC Genom..

[42] Wang J., Wen S., Symmans W.F., Pusztai L., Coombes K.R. (2009). The bimodality index: A criterion for discovering and ranking bimodal signatures from cancer gene expression profiling data. Cancer Inform..

[43] Tibshirani R., Hastie T. (2007). Outlier sums for differential gene expression analysis. Biostatistics.

[44] Justino J.R., Reis C.F.D., Fonseca A.L., Souza S.J., Stransky B. (2021). An integrated approach to identify bimodal genes associated with prognosis in cancer. Genet. Mol. Biol..

[45] Pascual-Ahuir A., Fita-Torro J., Proft M. (2020). Capturing and Understanding the Dynamics and Heterogeneity of Gene Expression in the Living Cell. Int. J. Mol. Sci..

[46] Yamada A., Kubo K., Takeshita T., Harashima N., Kawano K., Mine T., Sagawa K., Sugamura K., Itoh K. (1999). Molecular cloning of a glycosylphosphatidylinositol-anchored molecule CDw108. J. Immunol..

[47] Reilkoff R.A., Peng H., Murray L.A., Peng X., Russell T., Montgomery R., Feghali-Bostwick C., Shaw A., Homer R.J., Gulati M. (2013). Semaphorin 7a+ regulatory T cells are associated with progressive idiopathic pulmonary fibrosis and are implicated in transforming growth factor-beta1-induced pulmonary fibrosis. Am. J. Respir. Crit. Care Med..

[48] Garcia-Areas R., Libreros S., Iragavarapu-Charyulu V. (2013). Semaphorin7A: Branching beyond axonal guidance and into immunity. Immunol. Res..

[49] Ma B., Herzog E.L., Lee C.G., Peng X., Lee C.M., Chen X., Rockwell S., Koo J.S., Kluger H., Herbst R.S. (2015). Role of chitinase 3-like-1 and semaphorin 7a in pulmonary melanoma metastasis. Cancer Res..

[50] Zhang S., Kong F., Zheng L., Li X., Jia L., Yang L. (2024). SEMA7A as a Novel Prognostic Biomarker and Its Correlation with Immune Infiltrates in Breast Cancer. Int. J. Gen. Med..

[51] Elder A.M., Tamburini B.A.J., Crump L.S., Black S.A., Wessells V.M., Schedin P.J., Borges V.F., Lyons T.R. (2018). Semaphorin 7A Promotes Macrophage-Mediated Lymphatic Remodeling during Postpartum Mammary Gland Involution and in Breast Cancer. Cancer Res..

[52] Wang Y., Li C., Qi X., Yao Y., Zhang L., Zhang G., Xie L., Wang Q., Zhu W., Guo X. (2022). A Comprehensive Prognostic Analysis of Tumor-Related Blood Group Antigens in Pan-Cancers Suggests That SEMA7A as a Novel Biomarker in Kidney Renal Clear Cell Carcinoma. Int. J. Mol. Sci..

[53] Fu Y., Sun S., Bi J., Kong C. (2020). Construction of a risk signature for adrenocortical carcinoma using immune-related genes. Transl. Androl. Urol..

[54] Crump L.S., Wyatt G.L., Rutherford T.R., Richer J.K., Porter W.W., Lyons T.R. (2021). Hormonal Regulation of Semaphorin 7a in ER(+) Breast Cancer Drives Therapeutic Resistance. Cancer Res..

[55] Parkash J., Messina A., Langlet F., Cimino I., Loyens A., Mazur D., Gallet S., Balland E., Malone S.A., Pralong F. (2015). Semaphorin7A regulates neuroglial plasticity in the adult hypothalamic median eminence. Nat. Commun..

