# Supplementary material for: Bimodal Genomic Approach Predicting Semaphorin 7A (SEMA7A) as Prognostic Biomarker in Adrenocortical Carcinoma
Source: Cancers (Basel). 2025 Jun 21;17(13):2078. doi: 10.3390/cancers17132078 (PMC12249455; doi:10.3390/cancers17132078)
Supplement: Supplementary file 1 [file cancers-17-02078-s001.zip › Supplimentary_figures.pdf]

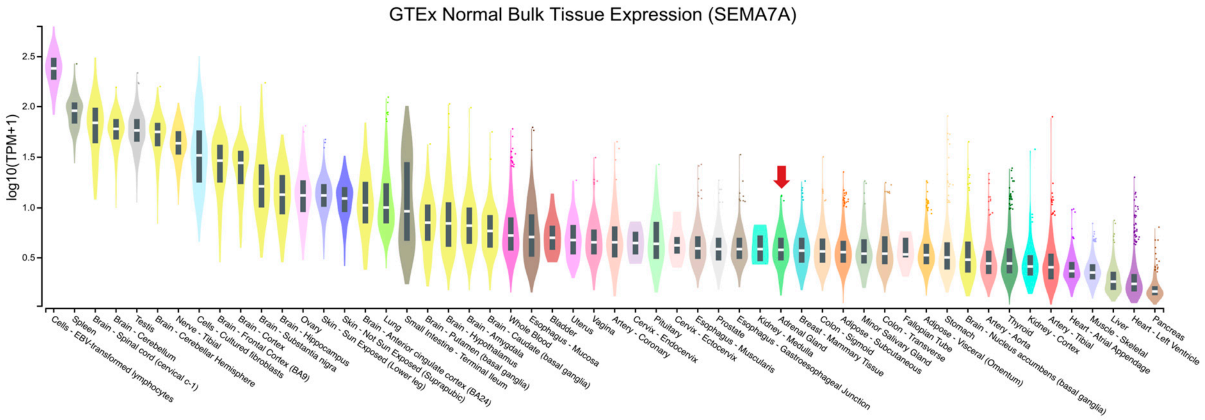

Figure S1: Distribution of SEMA7A expression across a wide range of normal human tissues obtained from GTEx database.

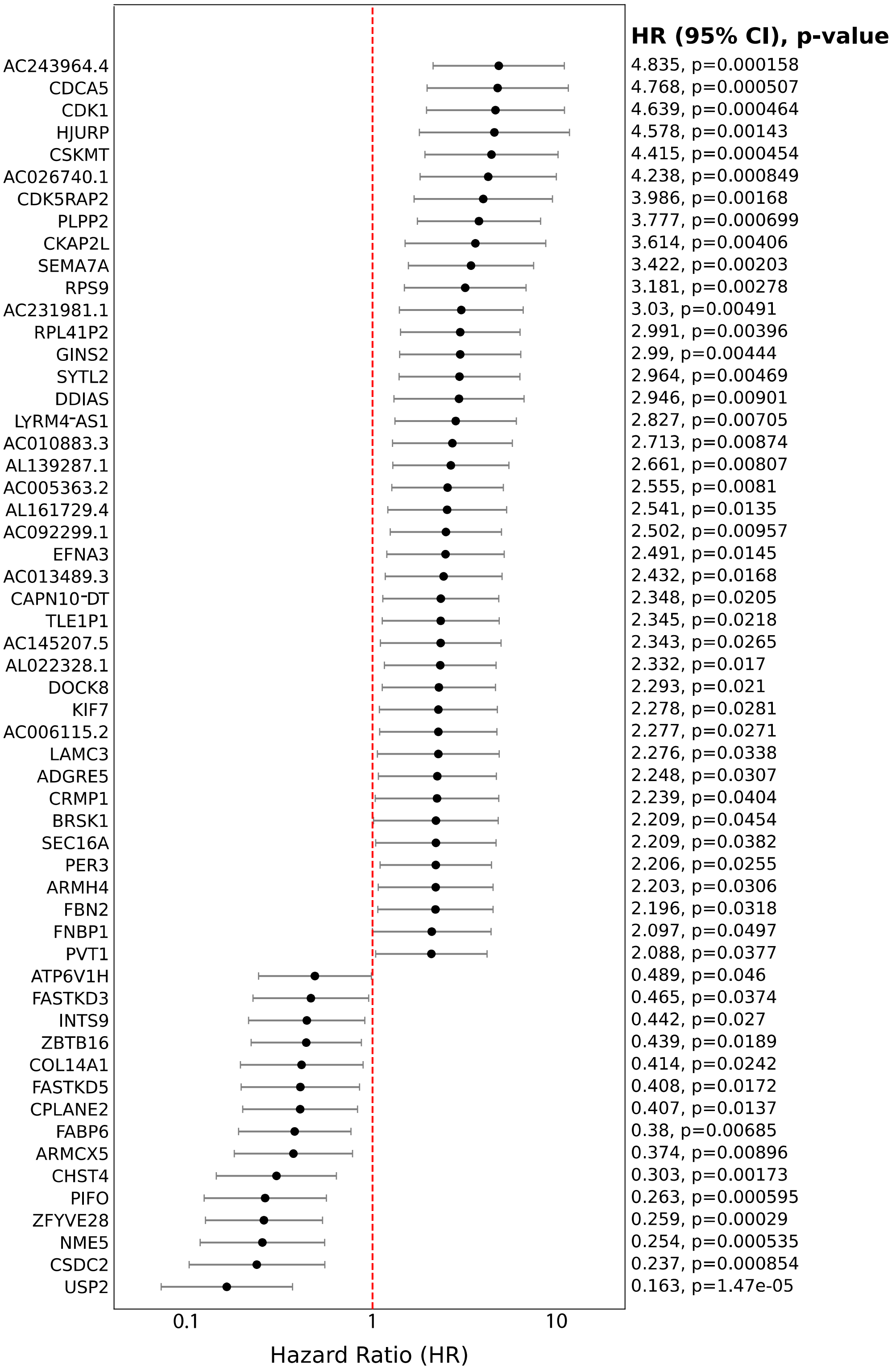

Figure S2: Forest plot of multivariate survival analysis of bimodal genes, adjusted for clinical covariates (Age, tumor stage and gender)
